# Tuberculosis-Associated Septic Shock: A Case Series

**DOI:** 10.7759/cureus.23259

**Published:** 2022-03-17

**Authors:** Veerendra Arya, Amarendra K Shukla, Brahma Prakash, Jitendra K Bhargava, Akriti Gupta, Brij B Patel, Pawan Tiwari

**Affiliations:** 1 General Medicine, School of Excellence in Pulmonary Medicine, Netaji Subhash Chandra Bose Medical College, Jabalpur, IND; 2 Pulmonary, Critical Care and Sleep Medicine, School of Excellence in Pulmonary Medicine, Netaji Subhash Chandra Bose Medical College, Jabalpur, IND; 3 Respiratory Medicine, School of Excellence in Pulmonary Medicine, Netaji Subhash Chandra Bose Medical College, Jabalpur, IND; 4 Physiology, School of Excellence in Pulmonary Medicine, Netaji Subhash Chandra Bose Medical College, Jabalpur, IND

**Keywords:** tb presenting as shock, tbss, tuberculosis septic shock, intensive care, tuberculosis

## Abstract

Tuberculosis septic shock (TBSS) is a rare diagnosis due to inherent diagnostic difficulty or attribution to alternate causes. We report six cases of TBSS, along with comorbidities, clinical characteristics, hospital course, and in-hospital outcomes. All patients were middle-aged, with a median age of 54.5 years (interquartile range (IQR): 47-62). Four patients were males, whereas two were females. Majority (n = 4, 66.7%) of patients had comorbidities. Diabetes mellitus (n = 3, 50%), systemic hypertension (n = 2, 33.3%), and chronic obstructive pulmonary disease (n = 1, 16.7%) were the reported comorbidities in included patients. Median Acute Physiology and Chronic Health Evaluation (APACHE) II score at admission was 12 (IQR: 12-16). All patients had a microbiologic diagnosis of tuberculosis (TB). Four patients (66.7%) had respiratory secretions positive for *Mycobacterium tuberculosis* (MTB) by acid-fast bacilli (AFB) smear or cartridge-based nucleic acid amplification test (CBNAAT), two had sputum positivity, one had induced sputum positivity, whereas another had bronchoalveolar lavage specimen positive for MTB. One patient had lymph node aspirate positivity, and another had chest wall abscess positive for MTB. All had drug-sensitive TB. Five patients could be prescribed all four primary antitubercular drugs; one patient had deranged liver enzymes, requiring initiation of modified antitubercular therapy (ATT). Five patients were discharged successfully, whereas one patient died during the hospital stay. In-hospital mortality was 16.7%.

## Introduction

Sepsis, a dysregulated host response to infection, remains a significant cause of mortality worldwide. According to a recent Global Burden of Disease study (2017), sepsis accounted for 48.9 million cases and 11 million deaths, and approximately 20% of global deaths could be attributed to sepsis [1]. The majority of community-based data on the etiology of sepsis come from the developed world. Bacterial infections are the most commonly reported cause of sepsis. Of these, respiratory infections account for a significant proportion [2-4]. Tuberculosis (TB), with an estimated disease burden of 2.64 million and nearly 1.5 million yearly deaths, remains the leading cause of death due to infectious diseases worldwide [5]. Around 3.4% of patients with TB require critical care [6]. However, shock due to TB, i.e., TB septic shock (TBSS), remains a rarely reported entity [7]. TBSS is diagnosed in cases presenting with systemic inflammatory response syndrome, clinicoradiologic or microbiologic diagnosis of TB, and exclusion of alternate etiology of sepsis or shock. This may be due to difficulty in diagnosing TBSS or attribution to alternate etiology. Because TBSS is such a rare occurrence, diagnosis requires a high index of suspicion. Herein, we report six cases of TBSS, along with clinical characteristics, laboratory findings, hospital course, and short-term outcome, and outline the challenges of managing TBSS patients.

## Materials and methods

Ours is a tertiary care hospital in central India, with dedicated pulmonary medicine services including TB wards and intensive care services. In the last six months, we admitted six patients who presented with features of systemic inflammatory response syndrome and who had clinicoradiologic features of TB. All these patients were treated as per standard guidelines for septic shock and were worked up for the causes. We did ultrasonographic evaluations to ascertain inferior vena cava (IVC) diameter, IVC inspiratory collapsibility, pericardial effusion, cardiac contractility, and lower limb venous Doppler to rule out other causes of shock. All patients were subjected to baseline investigations including liver function tests, kidney function tests, serum electrolytes, arterial blood gas analysis, serum lactate, procalcitonin levels, and chest radiograph. Blood, urine, and respiratory secretions were sent for bacterial cultures in all cases. Advanced radiologic investigations were done as indicated. Respiratory samples, pleural aspirates, and lymph node aspirates were evaluated for TB. These included acid-fast bacilli (AFB) smear, cartridge-based nucleic acid amplification test (CBNAAT), and liquid culture (mycobacterial growth indicator tube (MGIT)). Fine needle aspirates were sent for cytopathologic evaluation as indicated. All patients were initiated on antitubercular therapy (ATT) as per physician discretion and microbiologic or pathologic diagnosis of TB. Clinicodemographic details, comorbidities, initial presentation, radiologic features, hospital course, and short-term outcomes were described for all patients.

## Results

Case 1

A 53-year-old lady, a homemaker, was admitted to our hospital with complaints of fever for eight months, cough with sputum for one month, and progressive shortness of breath for the past two weeks. She also had an unintentional weight loss of around 15 kg in the last three months. There was no history of hemoptysis, paroxysmal nocturnal dyspnoea, or orthopnoea. She did not disclose any addiction or close contact with TB patients. However, she had been a known case of type 2 diabetes mellitus for the past eight months and was on regular treatment with oral hypoglycemic drugs, with reasonable blood sugar control (glycosylated hemoglobin (HbA1c): 7.1%). On examination, she was conscious, oriented, and afebrile. She had tachycardia (heart rate of 120/min), her blood pressure (BP) was 60/30 mmHg, respiratory rate was 20 per minute, and oxygen saturation (Spo2) on room air was 97%. On examination, she had a fluctuant non-tender swelling on the left side of her chest, without any change in size with respiration; findings were suggestive of left-sided pleural effusion with empyema necessitans. Other systemic examinations were unremarkable. Chest X-ray showed left upper zone consolidation with cavitation and left-sided loculated pleural effusion. She had anemia, predominantly neutrophilic leucocytosis (total leukocyte count (TLC): 12,400/microliter, with 85% neutrophils). Her platelet count, prothrombin time, renal functions, liver functions, and serum electrolytes were within normal limits. Blood sugars at admission were 300 mg/dl. There was no acidosis, and urine ketones were negative. Serum lactate was 6 mmol/liter. Serum procalcitonin was 0.2 ng/ml. On bedside ultrasound, IVC diameter was 18 mm, with significant collapsibility and normal cardiac output. On thoracic ultrasound, there was evidence of loculated left pleural effusion. Given these findings, she was promptly initiated on management on the lines of septic shock. She was administered 30 ml/kg of crystalloid fluid bolus. Still, she required inotropic support (noradrenaline 0.15 microgram/kg/min) to attain a target mean arterial pressure of 65 mmHg. She was started on piperacillin-tazobactam and levofloxacin; blood, urine, and pleural aspirate samples were simultaneously sent for microbiologic workup. Short-acting insulin, thromboprophylaxis, and supportive treatment were initiated. Pus aspirated from the left chest wall swelling was sent for gram stain, bacterial culture, AFB staining, CBNAAT, and TB culture (MGIT). Because of the strong clinical possibility of TB, she was also initiated on ATT with rifampicin, isoniazid, pyrazinamide, and ethambutol, according to body weight.

**Figure 1:**
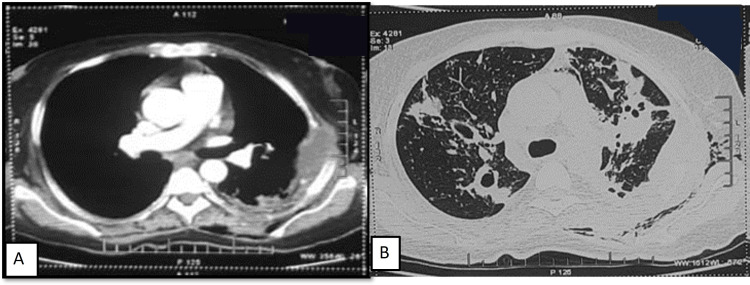
(A) Contrast-enhanced chest CT (mediastinal window) showing left lower lobe empyema with rib erosion and subcutaneous extension. (B) On lung window, bilateral upper lobe and right lower lobe infiltrates along with cavitation are seen.

She was further worked up for comorbidities and alternate etiology of shock. Two-dimensional (2D) echocardiography (ECHO) showed a 60% ejection fraction, no regional wall motion abnormality, and no valvular abnormality. Pus aspirated from chest wall lesion came positive for AFB. CBNAAT was positive for *Mycobacterium tuberculosis* (MTB); rifampicin resistance was not detected. Contrast-enhanced chest CT (Figures 1A, 1B) revealed collapse consolidation changes with the thick-walled cavity in bilateral upper lobes and superior segment of the left lower lobe. Chest CT also showed loculated left pleural effusion with thick enhancing walls, erosion of left second to fifth ribs, and soft tissue component extending into the chest wall, suggestive of empyema thoracis and necessitans.

The patient was gradually weaned off inotropic support over five days. Intravenous antibiotics were stopped after seven days, and she was discharged on day 12 of admission on ATT.

Case 2

A 56-year-old male, a farmer by occupation, presented with productive cough and fever for two months and progressive dyspnea for one month. He also had a history of losing appetite and weight (approximately 10 kg) over the past two months. There was no history of paroxysmal nocturnal dyspnoea or orthopnea. He was a known case of diabetes mellitus and systemic hypertension for two years and was on regular treatment. On admission, the patient was drowsy (Glasgow Coma Scale, GCS score: 12), had tachycardia (132/min), hypotension (BP of 70/40 mmHg), and tachypnea (respiratory rate of 36/min). His Spo2 on room air was 85%. The random blood sugar level was 212 mg/dl at admission. His laboratory investigation revealed a hemoglobin level of 8.6 g/dl, TLC of 11,500, and platelets level of 426,000. Kidney function and serum electrolytes were normal; liver enzymes were elevated (aspartate aminotransferase (AST): 312 units/liter; alanine transaminase (ALT): 450 units/liter); and bilirubin was normal. An electrocardiogram (ECG) was suggestive of sinus tachycardia. On chest X-ray, left upper zone consolidation with cavitation was seen, along with left-sided hydropneumothorax. The patient was immediately initiated on oxygen supplementation (5 liters per minute via nasal prongs). Bedside ultrasound showed low IVC volume, 75% collapsibility with inspiration, and normal cardiac contractility. The patient was given 30 ml/kg crystalloid fluid bolus but required noradrenaline to achieve target mean arterial pressure. We simultaneously started the patient on broad-spectrum antibiotics, including piperacillin-tazobactam, and modified ATT, i.e., levofloxacin and ethambutol.

**Figure 2:**
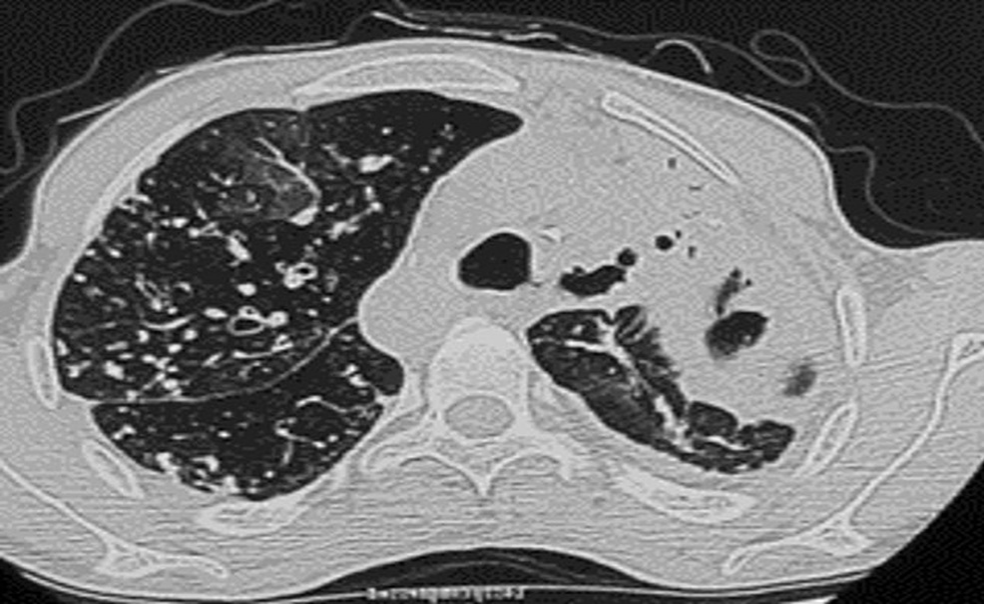
Chest CT showing left upper lobe collapse consolidation along with cavitation.

An intercostal drainage tube was inserted in view of hydropneumothorax; 300 ml pus was aspirated. Cultures from urine, blood, and sputum were all sterile. Chest CT showed left upper lobe collapse consolidation with cavitation (Figure 2). Sputum AFB was positive (3+), CBNAAT was positive, and rifampicin resistance was not detected. Echocardiography did not reveal any abnormality. His morning serum cortisol levels were normal (17 mcg/dl). Workup for acute viral hepatitis was negative. Over eight days, the patient was gradually weaned off inotropic support and oxygen support. Liver enzymes normalized over the next five days, and the patient was initiated on four-drug ATT (rifampicin, isoniazid, pyrazinamide, and ethambutol) on day nine of admission. He was subsequently discharged on day 15 on ATT.

Case 3

**Figure 3:**
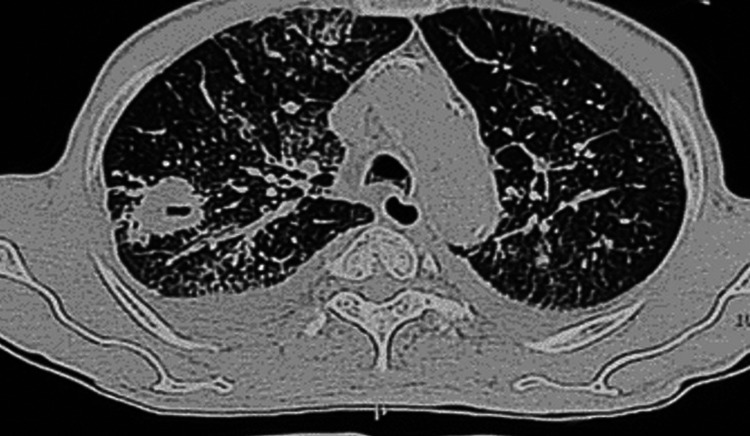
Chest CT showing right upper lobe cavitation along with bilateral lung nodules. Right moderate and left mild pleural effusion is also seen.

A 47-year-old male, a truck driver by occupation and chronic smoker (20 pack-years), presented to the emergency department with a three-week history of cough with sputum, fever, weight loss of approximately 5 kg, and loss of appetite. He was the household contact of a patient with active pulmonary TB. On admission, he was malnourished (BMI of 17.5 kg/m2). He had tachypnea (respiratory rate of 22/min), tachycardia (heart rate of 124/min), and hypotension (BP of 80/50 mm Hg). Bedside ultrasound showed narrow and collapsing IVC without any evidence of cardiogenic shock or alternate etiology. X-ray showed right upper zone consolidation with cavitation, along with right pleural effusion. Chest CT showed right upper lobe nodules with cavities along with right pleural effusion (Figure 3). He was immediately started on piperacillin-tazobactam and azithromycin. For management of septic shock, he was given fluid resuscitation, along with inotropic support.

Meanwhile, blood investigations showed TLC of 12,500/µl, platelets of 18,000/µl, and hemoglobin level of 8.9 g/dl; liver function test and kidney function test were within normal limits. The diagnostic pleural fluid evaluation showed pH of 7.3, glucose level of 78 mg/dl, and was exudative and predominantly neutrophilic; gram stain and AFB smear were negative. Vasopressin was added to noradrenaline because of persisting shock. After sending baseline serum cortisol levels, he was also concomitantly initiated on low-dose hydrocortisone. Because of persistent respiratory distress and hemodynamic instability, he was intubated and started on low tidal volume (6 ml/kg) lung protective ventilation as per acute respiratory distress syndrome (ARDS) protocol. Blood, endotracheal aspirate, pleural fluid, and urine were sent for bacterial culture, which was negative. Endotracheal aspirate AFB smear was positive, CBNAAT was positive for MTB, and rifampicin resistance was not detected. The patient was started on ATT on day two of admission; the rest of the treatment was continued. However, the patient developed refractory shock and died on day four of hospitalization.

Case 4

**Figure 4:**
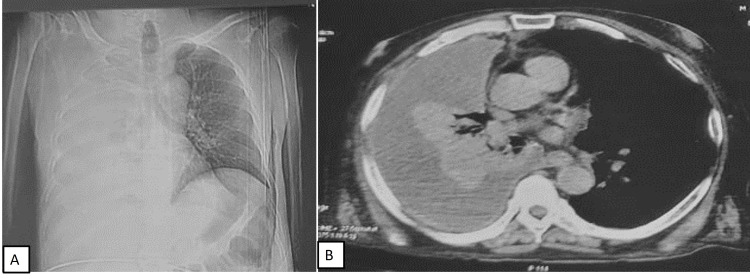
Chest radiograph (A) and chest CT (B) showing right-sided pleural effusion with passive collapse.

A 62-year-old male presented with progressive shortness of breath for two months along with a cough with scant mucoid expectoration for 10 days. He was on medication for type 2 diabetes mellitus and systemic hypertension for one year. On admission, the patient was conscious, oriented, and afebrile. His heart rate was 128/min, BP was 80/50 mmHg, Spo2 at room air was 98%, and the respiratory rate was 20/minute, without any accessory muscle use. On physical examination, a nontender, firm, and mobile left supraclavicular lymph node (2 x 2 cm) was palpable. On chest examination, a dull note was elicited on the entire right hemithorax, with absent air entry; examination of other organ systems was unremarkable. Blood sugar at admission was 168 mg/dl. Venous gases did not reveal any abnormality. On laboratory investigations, hemogram, liver, and kidney function tests were within normal limits. ECG showed sinus tachycardia. Chest X-ray showed a right-sided white-out lung (Figure 4A). The patient was immediately initiated on fluid resuscitation and subsequent inotropic support with low dose noradrenaline; intravenous antibiotics (ceftriaxone and azithromycin) were started. Oxygen supplementation was started at 3 liters per minute via nasal prongs. Therapeutic pleural fluid aspiration was done under ultrasound guidance; pleural fluid investigations were sent for infective workup, adenosine deaminase (ADA), and cytologic examination. Cervical lymph node aspiration was sent for cultures, AFB, CBNAAT, and cytology. Chest CT showed left-sided gross pleural effusion with the collapse of the left lung (Figure 4B). Lymph node cytopathology showed necrosis with granulomas; aspirate CBNAAT was positive for MTB and rifampicin resistance was not detected. He was started on ATT on day two of admission. His pleural fluid was exudative (protein 4.8 g/dl), lymphocytic (90%), and ADA was 66 U/l. On day four of admission, inotropes were stopped. The patient was subsequently discharged home on ATT on day nine of admission.

Case 5

**Figure 5:**
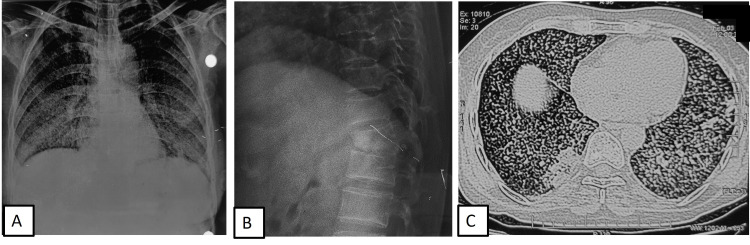
(A) Chest radiograph showing bilateral middle and lower zone nodules. (B) X-ray lateral view showing lower thoracic vertebra (D12) involvement. (C) Chest CT showing bilateral random nodules with focal consolidation in the right lower lobe.

A 45-year-old lady, a homemaker, presented to the outpatient department with fever and dry cough of one-month duration. She also had dyspnea on exertion for one week. There was no history of orthopnea or paroxysmal nocturnal dyspnea. She also had acute onset back pain in the lower thoracic area without any radiation, limb weakness, bladder, or bowel complaints. There was no significant history or comorbidities. On examination, GCS was normal. She had tachycardia (heart rate of 132/min), tachypnea (28/min), hypotension (BP of 60/40 mmHg), and hypoxia (SpO2 92% on room air). She also had pallor; there was no palpable peripheral lymphadenopathy. Bedside USG showed low volume collapsing IVC, normal cardiac functions, and no pericardial or pleural effusion. She received oxygen supplementation at the rate of 4 L/min via nasal prongs, fluid resuscitation, and inotropic support with noradrenaline. She was also started on antibiotics ceftriaxone and azithromycin. Chest X-ray showed bilateral middle and lower zone predominant nodular opacities (Figure 5A); X-ray of the spine showed D12 vertebral involvement (Figure 5B). Chest CT showed bilateral random nodules with lower lobe patchy consolidation (Figure 5C). Due to a clinicoradiologic diagnosis of disseminated TB, she was also started on four-drug ATT. Baseline investigations revealed normocytic normochromic anemia, hypoalbuminemia, and neutrophilic leucocytosis. Serum electrolytes, liver functions, and kidney functions were within normal limits. Induced sputum CBNAAT was positive for MTB; rifampicin resistance was not detected. Chest CT showed bilateral random nodules and patchy consolidation in bilateral lower lobes. Fundus examination was normal. By day three of admission, she was off oxygen and inotropic support and was discharged on day eight of hospitalization.

Case 6

**Figure 6:**
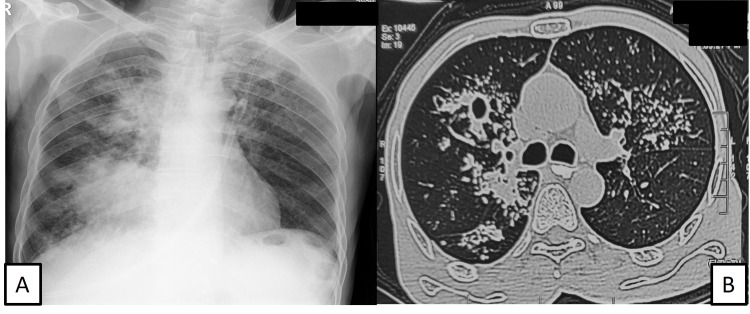
(A) Chest radiograph showing right upper zone and middle zone consolidation. (B) Chest CT showing bilateral upper lobe centrilobular nodules and right upper lobe cavitation with consolidation.

A 62-year-old male, a laborer by occupation and an ex-smoker (20 pack-years), was admitted with complaints of progressive breathlessness (modified Medical Research Council (mMRC) II) for one month. He also had a cough with scant mucoid expectoration for one month. He had lost around 10 kg weight in the last six weeks. There were no associated comorbidities or significant past history. On presentation, he was conscious, oriented, and afebrile. His heart rate was 130/min, respiratory rate was 18/min, saturation on room air was 98%, BP was 70/50 mmHg, and pulse was low volume but regular. General and systemic physical examination was unremarkable. Bedside ultrasound showed a small diameter collapsing IVC, with normal cardiac contractility. After fluid resuscitation, the patient had to be initiated on low dose noradrenaline to maintain mean target arterial pressure. He was started on broad-spectrum antibiotics ceftriaxone and azithromycin. Chest X-ray (Figure 6A) showed right upper and middle zone consolidation. CT of the chest showed bilateral upper lobe nodular infiltrates, small air-filled cavities, and right middle lobe collapse consolidation (Figure 6B). 2D ECHO did not reveal any abnormality; serum cortisol levels were normal. Empirical ATT was initiated because of radiologic findings; induced sputum was sent for microbiologic workup. Induced sputum CBNAAT was negative. On day two of admission, the patient required minimal inotropic support. Flexible bronchoscopy was done, which did not show any endobronchial lesion; BAL from the right middle lobe was positive for AFB, CBNAAT came positive for MTB, and rifampicin resistance was not detected. After one week of hospital stay, the patient got discharged on appropriate ATT.

Important details of all cases are tabulated in Table 1.

**Table 1:** Clinical data, laboratory investigations, and outcome of included patients with TBSS (n = 6). TB: tuberculosis; TBSS: TB septic shock; PaO2/FiO2: partial pressure of oxygen/fraction of inspired oxygen; APACHE: Acute Physiology and Chronic Health Evaluation; CBNAAT: cartridge-based nucleic acid amplification test; TLC: total leukocyte count; COPD: chronic obstructive pulmonary disease; AFB: acid-fast bacilli; DSTB: drug-sensitive tuberculosis.

Parameters	Case 1	Case 2	Case 3	Case 4	Case 5	Case 6
Age/sex	53/female	56/male	47/male	62/male	45/female	62/male
HIV serology	Negative	Negative	Negative	Negative	Negative	Negative
Comorbidities	Diabetes mellitus	Diabetes mellitus, Systemic Hypertension	COPD	Diabetes mellitus, systemic hypertension	None	None
Heart rate (per min)	120	132	124	128	132	130
Blood pressure systolic/diastolic (mmHg)	60/30	70/40	80/50	80/50	60/40	70/50
Respiratory rate (per minute)	20	36	22	20	28	18
PaO2/FiO2 ratio	>300	220	>300	>300	230	>300
Glasgow Coma Score	15	11	15	15	15	15
APACHE II score at admission	12	16	18	12	12	9
Mode of diagnosis of TB	Pus from empyema CBNAAT positive	Sputum AFB smear and CBNAAT positive	Sputum AFB smear and CBNAAT positive	Lymph node CBNAAT positive	Induced sputum CBNAAT positive	Bronchoalveolar lavage AFB smear and CBNAAT positive
TB drug sensitivity (CBNAAT)	DSTB	DSTB	DSTB	DSTB	DSTB	DSTB
Involved Organ Involvement	Lung, pleura and chest wall (empyema necessitans)	Pulmonary, pleural (hydropneumothorax)	Pulmonary	Disseminated, pleural, and lymph node	Disseminated; lung and musculoskeletal	Pulmonary
pH	7.36	7.48	7.2	7.4	7.45	7.38
Serum lactate (mmol/liter)	6	10	8	12	14	10
Serum procalcitonin	0.2	0.8	0.18	1	0.6	2
TLC (/microliter)	12,400	11,500	12,500	11,000	13,300	11,900
Differential leucocyte count (neutrophils, N%, lymphocytes, L%)	85%, 5%	88%, 6%	91%, 4%	84%, 9%	91%, 5%	89%, 7%
C-reactive protein (mg/liter)	50	70	66	48	60	42
Blood cultures	Sterile	Sterile	Sterile	Sterile	Sterile	Sterile
Urine cultures	Sterile	Sterile	Sterile	Sterile	Sterile	Sterile
Serum cortisol (microgram/deciliter)	16	17	24	18	21	20
Outcome	Discharged	Discharged	Died on day 7	Discharged	Discharged	Discharged

## Discussion

We describe herein six cases of TB presenting as septic shock, challenges faced in diagnosis and management, and short-term outcomes in these patients.

Sepsis and septic shock continues to be one of the most important causes of morbidity and mortality worldwide [1]. Yet, TB remains a rarely reported etiology of septic shock. Amongst a pool of 5,419 admitted patients across the globe, Kethireddy et al. were able to identify TBSS as the cause of septic shock in only 1% of the cases [8]. In a recent systematic review of 35 studies involving 1,815 hospitalized TB patients, 3.4% (95% CI: 1.6-5.7%) patients required ICU admission, with 48% in-hospital mortality. Sepsis or septic shock was described only in a few retrospective studies in this systematic review. In most cases, sepsis was either hospital-acquired or not specified due to TBSS [9-14]. Though shock was one of the indications for ICU admission in this systematic review, only TB-ARDS was an independent predictor for in-hospital mortality with an odds ratio of 3.9 (95% CI: 1.7-8.7) [6]. In their retrospective study including 212 patients with TB requiring ICU admission, Thomas et al. [15] reported sepsis as the cause of admission in 26 (12.3%) cases. Also, 67 patients (31.6%) required vasopressors, of whom 62 (92.5%) had a warm shock. In this study, pulmonary TB (OR: 2.83; 95% CI: 1.15-6.95) and vasoactive treatment (OR: 15.8, 95% CI: 6.4-39.2, p < 0.001) were independent indicators of ICU mortality [15].

TBSS is more common in adults than children [16]. TB in immunocompromised patients can manifest with systemic inflammatory response syndrome and multiple organ dysfunctions and present with sepsis or septic shock [8]. Gachot et al. described four individuals with disseminated TB who suffered septic shock. *M. tuberculosis* was isolated from blood using the isolator centrifugation method [17]. Ahuja et al. also described two patients with HIV and TB risk factors with septic shock-like hemodynamic characteristics, including a high cardiac output and low systemic vascular resistance. Both patients died within two weeks, and TB was discovered at autopsy [18]. Furthermore, Vadillo et al. reported the instance of a similar patient whose disease was confirmed as TB on day two of hospitalization depending on the outcomes of bronchial and bone marrow biopsies; this patient began treatment immediately but died on day three of hospitalization [19]. In another study, including HIV patients with TB, a low CD4 (cluster of differentiation 4) cell count (<200/µL), renal dysfunction, diffuse parenchymal disease, miliary pattern, and untreated TB were associated with increased mortality [20].

In our case series, even though none of the patients tested positive for HIV or had any overt immunocompromised status, they all had a septic shock at presentation. The mortality risk factors for critically unwell TB patients have been found [6,10]. There is a lot of information to support the TBSS case fatality [8]. It is indisputable that early detection of septic shock and fluid resuscitation along with appropriate antibiotic therapy reduce mortality. However, acute presentation and overlapping features with community-acquired pneumonia (CAP) or sepsis syndromes make the diagnosis of TBSS difficult [15,21,22]. To add to the problem, up to 7% of CAP can be due to TB itself [23]. Typical chest radiologic abnormalities contribute significantly to a high level of clinical suspicion. However, chest X-ray findings lack specificity and can be misdiagnosed as CAP [15,24]. These patients are too sick to be shifted for advanced imaging like CT or MRI. Appropriate antibiotic therapy in TBSS, i.e., ATT, is seldom started without sputum smear or CBNAAT reports, even though recommended by international guidelines (Infectious Diseases Society of America) [25].

An effective testing strategy should be quickly combined with a high clinical suspicion for TB to arrive at a conclusive diagnosis. The initial stage in diagnosis, sputum examination with acid-fast staining, is quick and easy, but it lacks reproducibility due to limited sensitivity [26]. Also, a significant proportion of patients have extrapulmonary TB, or sputum-negative pulmonary disease [6,15], thereby limiting microbiological diagnosis. Still, newer modalities like CBNAAT can help achieve diagnosis in most patients [15].

We could discharge five patients, and one died of septic shock despite appropriate therapy. Regarding workup for the cause of shock, we sent all recommended cultures and could not find an alternate etiology, except for TB. We measured serum cortisol levels in all the patients. We did 2D echocardiography in all the patients to rule out cardiogenic shock, which showed normal ejection fraction, no regional wall motion abnormality, or valvular defects. It is a well-known fact that early initiation of antibiotic therapy leads to lower mortality in septic shock. In our cases, we initiated early antitubercular treatment and broad-spectrum antibiotics to cover other causes of community-acquired sepsis. Our findings are limited due to the small sample size and lack of prospective evaluation. However, we evaluated all patients for possible alternate causes of sepsis and septic shock and achieved microbiological or histopathologic evidence of TB in all cases.

## Conclusions

TBSS remains underdiagnosed and underreported as a cause of septic shock, especially in TB endemic countries. Early diagnosis, fluid resuscitation, inotropic support, and appropriate antibiotic administration remain the cornerstone of management in TBSS, like any septic shock. A high index of suspicion for TB and prompt septic shock management are lifesaving in such cases. In patients with a high clinical likelihood of septic shock due to TB, ATT should not be withheld for a textbook microbiologic diagnosis. Clinicians should be vigilant of TB presenting as septic shock, especially in high endemic areas. Multicentric prospective observational studies are needed to identify the prevalence and factors predicting the outcome of TBSS to optimize the clinical care of these patients.
